# Isolated Mitochondrial Preparations and *In organello* Assays: A Powerful and Relevant *Ex vivo* Tool for Assessment of Brain (Patho)physiology

**DOI:** 10.2174/1570159X21666230303123555

**Published:** 2023-05-12

**Authors:** Faraz Ahmad, Siva Ramamorthy, Mohammed Y. Areeshi, Ghulam Md. Ashraf, Shafiul Haque

**Affiliations:** 1 Department of Biotechnology, School of Bio Sciences and Technology (SBST), Vellore Institute of Technology, Vellore, 632014, India;; 2 Medical Laboratory Technology Department, College of Applied Medical Sciences, Jazan University, Jazan, 45142, Saudi Arabia;; 3 Department of Medical Laboratory Sciences, College of Health Sciences, and Sharjah Institute for Medical Research, University of Sharjah, Sharjah, 27272, United Arab Emirates;; 4 Research and Scientific Studies Unit, College of Nursing and Allied Health Sciences, Jazan University, Jazan, 45142, Saudi Arabia;; 5 Gilbert and Rose-Marie Chagoury School of Medicine, Lebanese American University, Beirut, Lebanon;; 6 Centre of Medical and Bio-Allied Health Sciences Research, Ajman University, Ajman, United Arab Emirates

**Keywords:** Intrasynaptic mitochondria, density gradient centrifugation, mitochondrial membrane potential (MMP), electron transport chain (ETC), calcium capacitance, reactive oxygen species (ROS), glutathione (GSH), Trolox equivalent antioxidant capacity (TEAC)

## Abstract

Mitochondria regulate multiple aspects of neuronal development, physiology, plasticity, and pathology through their regulatory roles in bioenergetic, calcium, redox, and cell survival/death signalling. While several reviews have addressed these different aspects, a comprehensive discussion focussing on the relevance of isolated brain mitochondria and their utilities in neuroscience research has been lacking. This is relevant because the employment of isolated mitochondria rather than their *in situ* functional evaluation, offers definitive evidence of organelle-specificity, negating the interference from extra mitochondrial cellular factors/signals. This mini-review was designed primarily to explore the commonly employed *in organello* analytical assays for the assessment of mitochondrial physiology and its dysfunction, with a particular focus on neuroscience research. The authors briefly discuss the methodologies for biochemical isolation of mitochondria, their quality assessment, and cryopreservation. Further, the review attempts to accumulate the key biochemical protocols for *in organello* assessment of a multitude of mitochondrial functions critical for neurophysiology, including assays for bioenergetic activity, calcium and redox homeostasis, and mitochondrial protein translation. The purpose of this review is not to examine each and every method or study related to the functional assessment of isolated brain mitochondria, but rather to assemble the commonly used protocols of *in organello* mitochondrial research in a single publication. The hope is that this review will provide a suitable platform aiding neuroscientists to choose and apply the required protocols and tools to address their particular mechanistic, diagnostic, or therapeutic question dealing within the confines of the research area of mitochondrial patho-physiology in the neuronal perspective.

## 1 INTRODUCTION

Neuronal processes of synaptic transmission, membrane potential maintenance, and Ca^2+^ exchange/extrusion require high amounts of energy, making the brain among the most prominent sites of energy consumption in the body. Mitochondria, the cellular power houses, hence are critical mediators of neuronal physiology, fulfilling the high-energy demands of neurons. In addition, mitochondria regulate calcium and redox signalling, and cellular death through regulation of the apoptotic and necrotic signals [1-3]. In particular, the case of intrasynaptic mitochondria is very interesting. Owing to the high-energy demands associated with the processes of neuronal signalling and plasticity, synapses, the major sites of interneuronal communication are enriched in mitochondria. Recent studies have indicated that these synapse-localized mitochondria (synaptic mitochondria or SM) are significantly different from the free mitochondrial pool (although both share the same origin) in terms of physical sizes as well as proteome, lipidome, and enzyme profiles [4-6]. Moreover, SM are configured for rapid adaptive responses to match the dynamic changes associated with synaptic signalling and plasticity [7, 8]. Interestingly, SM are more vulnerable to insults such as redox and calcium dyshomeostasis (and swelling) than their free neuronal mitochondrial counterparts [5, 8-10]. For these reasons, recent studies, including from our group, have been primarily directed to the evaluation of synapse-specific mitochondria in pathophysiological brain states, ranging from early-life insults [11, 12] to ageing-induced neurodegeneration [13-15].

Given the peculiar and multifactorial roles of brain mitochondria, it is hardly surprising that dysfunction of mitochondria is implicated as a primary pathogenic mechanism in almost all brain pathologies [16-21]. A comprehensive understanding of the brain's mitochondrial functions both under normal conditions and in pathological states is warrantied for understanding the disease pathology, identification of biomarkers, and for the development of effective neuroprotective and disease-modifying therapies. While appreciable progress has been made in the previous decades, many aspects of mitochondrial functions (and dysfunction) unique to brain mitochondria have still remained obscure. In this regard, biochemically isolated viable mitochondrial preparations retain their compartment properties and provide a suitable platform for *in organello* (patho)physiological assessment in a mitochondrial-specific manner [22-24]. Indeed, there are clear advantages of using isolated mitochondria for the assessment of their roles in neuronal (patho)physiology. First, these preparations negate the interference from cellular signalling cascades and other factors that regulate mitochondrial function. Second, they allow a high-throughput and in-depth analysis of functions, minimizing time and sample-to-sample variation. Third, preparation of viable and metabolically active mitochondria is possible even from long-term stored (cryopreserved) tissues of human and rodent origin. This indeed constitutes a powerful experimental tool in clinical research involving post-mortem human brain tissue samples in view of the technical challenges associated with their analyses.

Since the late 1940s, when biochemical isolation of liver mitochondria was pioneered by the Palade lab [25], considerable progress has been made in the techniques for functional assessment of intact viable mitochondrial preparations from fresh and frozen brain tissues. This has in turn been instrumental in providing distinct information regarding mitochondrial function and dysfunction in diseased states [22, 26]. While there are several reviews delineating the relevance of mitochondrial biology from the perspective of neuroscience research, a comprehensive review of the methodological procedures and protocols for an *in organello* assessment of isolated brain mitochondria has been lacking. The purpose of this review is to assemble the commonly used protocols for mitochondrial isolation, their quality assessment, and functional characterization in a single place. While the authors do not claim to discuss all the *in organello* techniques for mitochondrial assessment, the present review does aim to cover the widely employed and relevant biochemical assays and will provide considerable information to aid neuroscience researchers in choosing and applying the selected protocols, tools, and citations to address a particular research question pertaining to mitochondrial patho-physiology in the neuronal health and disease. Efforts have also been made to emphasize protocols which require only the basic biochemical equipment commonly available in most life sciences laboratories, rather than specialized cost-incurring instruments. This has also allowed us to choose methodologies which are simple, systematic and efficient, and simultaneously time- and cost-efficient.

## 2 ISOLATION OF MITOCHONDRIAL PREPARATIONS, QUALITY ASSESSMENT AND CRYOPRESERVATION

### 2.1 Density Gradient Isolation of Brain Mitochondria

**Fig. (1):**
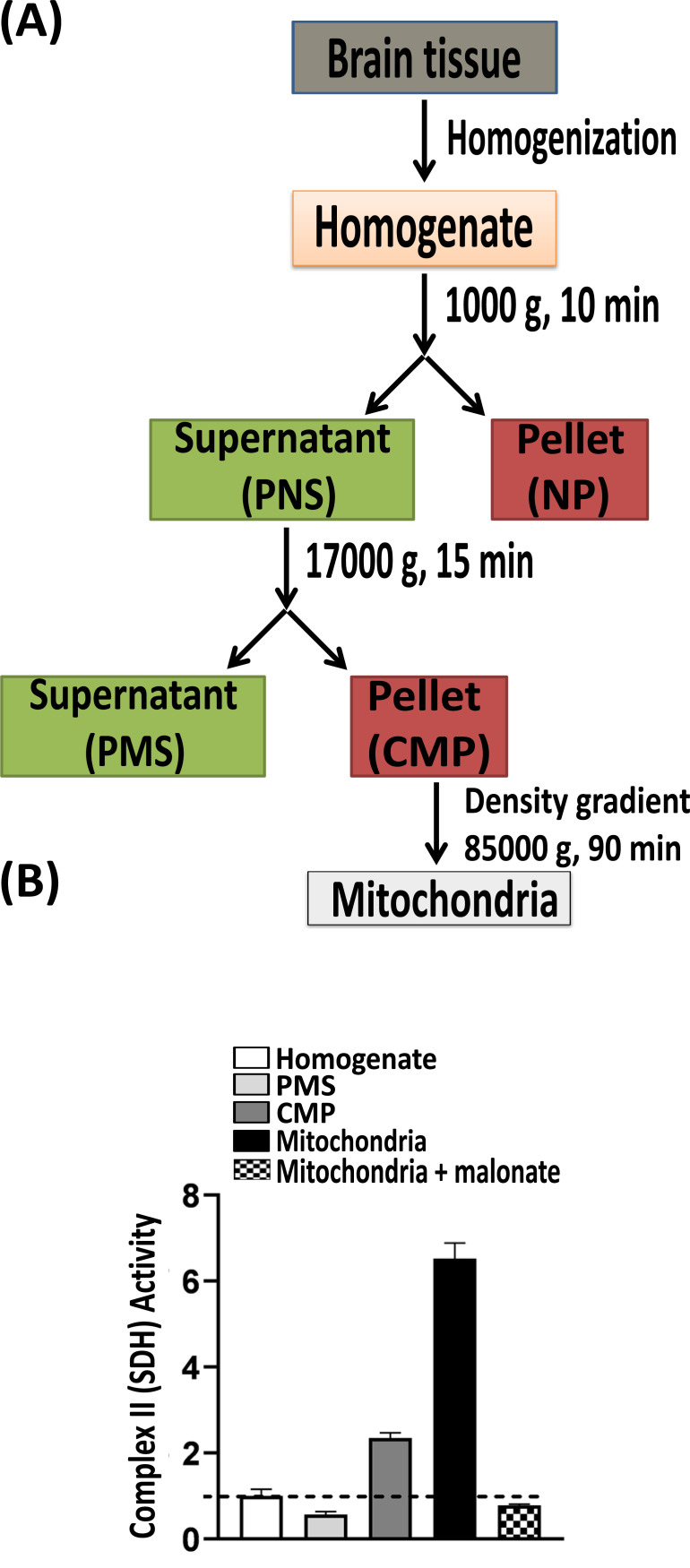
Biochemical isolation of brain mitochondria. (**A**) Flowchart reveals the basic protocol for isolation of mitochondria using an equilibrium density gradient sub-cellular fractionation protocol. The first slow speed centrifugation of the mechanically homogenized brain tissue sample results in a post nuclear supernatant (PNS) and a crude nuclear pellet (NP). Further high speed centrifugation of the PNS generates the crude mitochondrial pellet (CMP) and the cytosolic post mitochondrial supernatant (PMS). Pure free (nonsynaptic) mitochondria are separated from the CMP using density gradient centrifugation. (**B**) Assessment of enrichment (and integrity) of the mitochondrial fraction in comparison to the starting brain homogenate and other intermediate fractions can be performed using a spectrophotometric assay of succinate dehydrogenase (SDH) activity (section 2.2.7.1; [61]). Malonate, a competitive SDH inhibitor was used to establish the specificity of the assay.

Different protocols exist for the isolation of *ex vivo* preparations of viable mitochondria from the different starting materials (*e.g*., different tissue- and cell-type and organism). The basic principle however is similar and relies on a) tissue/cell homogenization and b) sequential centrifugation (Fig. **1**). Sequential centrifugation of the homogenate with increasing sedimentation forces enables a rough fractionation of the cellular environment. Thus, centrifugation of the brain homogenate at low speed (ca. 600-800 g) pellets down nuclei and cellular debris, and a second high-speed centrifugation step (ca. 7000-8000 g) generates a mitochondrial pellet. This crude mitochondrial pellet has sometimes been used *in organello* studies; however, it should be noted that this crude preparation contains appreciable extra-mitochondrial contaminants [24, 27]. Further isolation of high-purity enriched mitochondrial sample requires equilibrium density gradient centrifugation-based protocols which result in the acquirement of mitochondria as a focused layer at the isopycnic point in the gradient [27-29].

Density gradient centrifugation for mitochondrial isolation from rat liver was first proposed in the late 1940s [25] and the first protocols for brain mitochondria isolation were published in the 1960s [30, 31]. Since then, a number of specialized protocols have also been proposed for brain mitochondria isolation, employing different density gradient media; such as sucrose [32], Ficoll [11, 12, 33-35], and Percoll [27, 36-39]. It is relevant to note here that cryopreserved rodent and human tissues can be effectively used as a source of functionally and bioenergetically viable mitochondria [40-44]. Readers are directed to the specific protocols for primary neurons and astrocytes [28, 45, 46], rodent brain tissue [32, 37-39] and spinal cord [33, 36], and human brain tissues obtained post-mortem [42, 47, 48] and post-surgery [49, 50]. Specific protocols have also been designed to isolate viable and bioenergetically well-coupled mitochondria from neonatal rodent brains [51]. Crude mitochondria have also been isolated from small amounts of mouse brain tissue, which is very convenient for differential assessment of region-specific mitochondrial activity and viability [41].

Interestingly, subcellular fractionation of synaptic and non-synaptic brain mitochondria has also been used for comparative studies [12, 52], which has been instrumental in deducing the differences in the two mitochondrial populations in terms of bioenergetics profile, Ca^2+^, and redox regulatory roles and susceptibility to insults/stress [12, 35, 39, 53, 54]. Similar studies have also been carried out for differential characterization of neuronal and glial mitochondria [34]. It is interesting for the readers to note here that subcellular fractionation protocols also exist for isolation of membrane fraction containing mitochondria-associated membranes (MAM) [55-57], which are thought to be physically associated contact sites between endoplasmic reticulum membrane proteins and outer mitochondrial membrane. However, functional analyses of these preparations are beyond the scope of this review and will not be discussed here.

Lastly, other methods are also available for mitochondrial isolation. For example, immunopurification (and subsequent magnetic extraction) of brain mitochondria has been carried out using antibodies specific for outer mitochondrial membrane proteins, such as translocase of the outer membrane (TOM) complex component TOM22 [50]. Isolation of mitochondria by integrated zone electrophoresis has also been performed [58]; however, it requires specialized skills and instruments such as a free-flow apparatus.

### 2.2 Quality Control Procedures: Mitochondrial Yield and Integrity

#### 2.2.1 Electron Microscopy (EM)

EM is obviously the best and the most direct method to assess the enrichment and morphological intactness of *ex vivo* mitochondrial preparations. Free mitochondria are easily identifiable as ribbed structures with a clear double membrane [59, 60]. Time and cost constraints along with the requirement of specialized skills and equipment however limit the use of electron microscopy for the sole purpose of evaluation of mitochondrial purity and intactness.

#### 2.2.2 Mitochondrial Stains

We and others have employed Janus green B, a supravital lipophilic cationic stain for a quick and simple assessment of the enrichment, viability, and integrity of isolated mitochondria. Since the dye can only be taken up by metabolically active mitochondria with an intact membrane potential (see section 3.1), colorimetric [61, 62] or microscopic [63, 64] evaluation of Janus green B retention serves as a good indicator of the intactness of isolated mitochondria.

The enrichment of mitochondria (and determination of their mass) in the *ex vivo* preparations can also be evaluated using the fluorescent MitoTracker^®^ dyes [65, 66]. Similar to Janus green B, these potential-indicating cationic dyes accumulate electrophoretically into mitochondria. Unlike Janus green B, however, MitoTracker^®^ forms covalent adducts with the thiol groups of the matrix proteins, making their fluorescence independent of mitochondrial potential. Visualisation of mitochondria can be performed using either flow cytometry [67], fluorescence microscopy [68], or spectroscopy [44, 69].

#### 2.2.3 Marker Proteins: Immunoblotting and Activity Assays

Enrichment of mitochondria in the biochemically isolated preparations can be confirmed by immunoblotting (with respect to the starting homogenates) using antibodies against mitochondrial marker proteins [70]. Several candidates can be used for this purpose; such as electron transport chain complexes, TOM20, and voltage-dependent anion-selective channel (VDAC), *etc*. However, we strongly recommend using activity assays of marker proteins such as succinate dehydrogenase (see section 3.4.2; [11, 12, 61]) or citrate synthase (section 2.2.7.2) for determining the enrichment.

#### 2.2.4 Lactate Dehydrogenase (LDH; EC 1.1.1.27) Activity

LDH is a cytosolic enzyme and its absence is sometimes taken as an indicator of mitochondrial enrichment [46, 67]. Although it is not very often used for this purpose, it is worth mentioning it here because of the utility of the assay in determining the integrity of cells as well as isolated synaptic terminals [71, 72]. Briefly, the assay relies on spectrophotometric measurement of the oxidation of exogenously added NADH in the presence of an exogenous LDH substrate, pyruvate [73]. Similarly, absence of activity of a synaptic marker enzyme, acetylcholinesterase in mitochondrial preparations has sometimes been employed for confirmation of minimal synaptic contamination [46]. This is particularly relevant for the quality assessment of mitochondrial samples isolated from brain tissue samples.

#### 2.2.5 Cytochrome c Release

Similar to the LDH release assay, cytochrome c release assay is often employed to evaluate the intactness of isolated mitochondria [26]. Cytochrome c release from mitochondrial samples can be assessed by immunoblotting [70] or by evaluating the differential (under normal oxidized conditions, and in dithionite-treated reduced conditions) absorption spectra of the extracellular medium (supernatant) [74].

#### 2.2.6 Oxidation of Cytochrome c and Ascorbate

Several other assays have also been routinely used to assess the intactness of the outer mitochondrial membrane (OMM) in isolated mitochondrial preparations. The basic principle of all these methodologies are based upon the impermeability of OMM for certain chemicals (cytochrome c and ascorbate) or localization of certain enzymes in the mitochondrial intermembrane space (*e.g*. adenylate kinase; section 2.2.7.4). Evaluation of the KCN-sensitive (complex IV-mediated) oxidation of exogenously added cytochrome c (see section 3.4.5) has been employed as a measure of OMM intactness [75, 76]. Similarly, the ascorbate test for the integrity of OMM of mitochondrial preparations evaluates the oxidation of exogenous ascorbate. Monitoring ascorbate oxidation however, requires indirect polarographic evaluation of oxygen consumption using a Clark-type electrode (see section 3.5) in the presence and absence of N, N, N’, N’-tetramethyl-p-phenylenediamine (TMPD). The detailed procedure has been previously published [41, 77].

#### 2.2.7 Enzyme Activity Assays

*In organello* activity assays of several mitochondrial marker enzymes have been employed to evaluate both enrichment (yield) and integrity of mitochondrial preparations. We discuss below the most-widely used assays.

##### 2.2.7.1 Succinate Dehydrogenase (SDH; E.C. 1.3.5.1) Activity

As previously indicated, succinate dehydrogenase (complex II) is localized in the inner mitochondrial membrane and hence its activity serves as a good indicator of both enrichment and integrity of isolated mitochondria, as demonstrated by us and others [12, 61, 78]. Refer to section 3.4.2 for details of the colorimetric assay. Generally, the integrity of isolated mitochondrial preparations is thought to be good if 90-95% mitochondria are intact [12, 79].

##### 2.2.7.2 Citrate Synthase (CS; EC 2.3.3.1) Activity

The yield of mitochondria (*i.e*. the efficiency of the isolation protocol) can also be evaluated by measuring the activity of the mitochondrial marker enzyme, citrate synthase (CS) [80, 81]. The assay spectrophotometrically measures the reduction of 5, 5’-dithiobis(2-nitrobenzoic acid) (DTNB) to 5’-thionitrobenzoate (TNB^-^) anion in the presence of exogenous substrates, acetyl-CoA and oxaloacetate [41, 82]. Since CS is localized in the mitochondrial matrix, its activity in isolated mitochondria before and after membrane disruption by freeze-thaw cycles (2-3 cycles of quick freezing at -85°C and thawing at 4°C on ice) or by extraction with Triton X-100 is regarded as a good measure of the integrity of the inner mitochondrial membrane (IMM). CS activity is often also employed as a normalization control for the various *in organello* assays (section 7).

##### 2.2.7.3 Fumarase (E.C. 4.2.1.2) Activity

We and others have used activity assessment of another mitochondrial matrix enzyme, fumarase as an indicator of mitochondrial yield and integrity of its inner membrane [12, 77, 83]. The spectrophotometric assay follows the formation of fumarate upon addition of exogenous malate to isolated mitochondria [84]. Whether using CS or fumarase as the marker enzyme, the integrity of mitochondria can be expressed in %, calculated as [1-(γb/γa)]*100; where γb and γa are the enzyme activity rates before and after membrane disruption.

##### 2.2.7.4 Adenylate Kinase (AK; EC 2.7.4.3) and Glutamate Dehydrogenase (GDH; EC 1.4.1.3) Activities

*In organello* activity assays for adenylate kinase (AK) and glutamate dehydrogenase (GDH) are sometimes also employed to evaluate the intactness of mitochondrial OMM and IMM, respectively as the two enzymes are localized in mitochondrial intermembrane space and matrix, respectively [85].

#### 2.2.8 Calcium Buffering Capacity

Lastly, evaluation of mitochondrial calcium buffering capacity serves as a fast and efficient fluorimetric measure of the integrity of isolated mitochondria since the uptake of Ca^2+^ occurs through the inner membrane-localized mitochondrial calcium uniporter and is dependent on an intact mitochondrial membrane potential [27]. Refer to section 3.3 for details of the assay.

#### 2.3 Cryopreservation of Isolated Mitochondria

The time taken for mitochondrial isolation and the batch-to-batch variability may hinder high throughput assessment of their functions [86]. To improve assay efficiency and consistency, long-term storage of isolated mitochondria is recommended and can be performed using trehalose [87], dimethyl sulfoxide (DMSO; [88]), glycerol [89], or ethylene glycol [90]. These methods of cryopreservation of isolated mitochondria have been demonstrated to optimally preserve mitochondrial outer membrane integrity and bioenergetic functions. Nevertheless, we would like to emphasize here that wherever possible, *in organello* assays of mitochondrial functions should be performed freshly isolated samples.

## 3 MITOCHONDRIAL ACTIVITY AND BIOENERGETIC ASSAYS

### 3.1 Mitochondrial Membrane Potential (MMP or Δψ_m_)

Mitochondria use oxidizable substrates to generate an electrochemical proton gradient across the inner mitochondrial membrane. The direction of this mitochondrial membrane potential (Δψ_m_) is such that the inside is electronegative, and hence promotes inward transport of cations and forms the basis for ATP generation [65]. As such, Δψ_m_ is a robust indicator of mitochondrial integrity, activity and bioenergetic capacity. Indeed, any dysregulation of Δψ_m_ (whether low or persistently high) indicates possible functional and structural dysfunction of the mitochondria [2, 91].

Cationic lipophilic fluorescent dyes have been envisioned as excellent tools to measure MMP for a long time [65, 92-94]. For example, we used safranin O, a rhodamine derivative for the assessment of Δψ_m_ of isolated mitochondrial preparations at both the basal and energized states (in presence of substrates, malate and glutamate) [11, 12]. Basal Δψ_m_ can be simply measured by the amount of safranin O retained in the mitochondrial pellet. On the other hand, fluorescence quenching of safranin O after addition of energy substrates serves as a suitable measure of mitochondrial Δψ_m_ generation (*i.e*. Δψ_m_ at energized state) [11, 12, 41]. Moreover, safranin O can also be employed to deduce the absolute values of Δψ_m_ in isolated mitochondria with calibration using a K^+^ gradient [95, 96].

Other cationic fluorescent dyes have also been used for assessment of Δψ_m_ in isolated mitochondrial preparations; however 5, 5’, 6, 6’-tetrachloro-1, 1’, 3, 3’-tetraethylbenzi-midazolylcarbocyanine iodide (more commonly known as JC-1) which exhibits a green-to-red emission shift upon mitochondrial accumulation, has become the gold standard for measuring Δψ_m_ [67, 97-100]. Noteworthy, JC-1 or safranin O mediated Δψ_m_ measurements have been shown to be performed using fluorescence spectroscopy [11, 12, 54, 101], fluorescence microscopy [65], or fluorescence-activated cell sorting (FACS) [102].

### 3.2 Mitochondrial Permeability Transition (Induced Swelling)

Mitochondrial permeability transition (mPT) or the opening of the mitochondrial permeability transition pore (mPTP) is the sudden increase in the permeability of the inner mitochondrial membrane to solutes up to 1.5 kDa (which are normally impermeable) [103]. mPT is primarily caused by stressors such as calcium overload and oxidative stress and results in disruption of the proton gradient, uncoupling of respiration, cessation of ATP production, and bioenergetic failure. mPT ultimately leads to osmotic swelling and outer membrane rupture and signals cell death [104, 105]. Not surprisingly, vulnerability of mitochondria to Ca^2+^-induced swelling has been evaluated in several pathological states [36, 106-109]. Interestingly, recent evidences also suggest that transient mPTP opening may also be a regulator of brain development [110].

*In organello* induction of mPT in isolated mitochondria is done in the presence of high Ca^2+^ in the extracellular medium. This Ca^2+^-induced swelling can be monitored by light scattering at 520 nm, with a steep reduction in the absorbance at this wavelength; both under energized (in presence of substrates malate and glutamate) and de-energized states [36, 67, 70].

### 3.3 Calcium Capacitance (Buffering Capacity)

Calcium buffering capacity or calcium retention capacity of mitochondria is essential for cellular functions, particularly in neuronal systems for protection against excitotoxicity [111]. Importantly, intramitochondrial calcium is significant not only for intracellular buffering but also for metabolic regulation of many key mitochondrial enzymes (such as pyruvate dehydrogenase, α-ketoglutarate dehydrogenase, *etc*.) [2]. Calcium-sensitive fluorescent probes such as Fura 6F and Calcium Green 5N are excellent tools to monitor calcium uptake and release in isolated mitochondrial samples. Readers are advised to consult the fluorescence spectroscopy-based methodology described in several previous studies [27, 36, 54, 70, 74, 101, 112, 113].

### 3.4 Activities of the Electron Transport Chain (ETC) Complexes

**Fig. (2):**
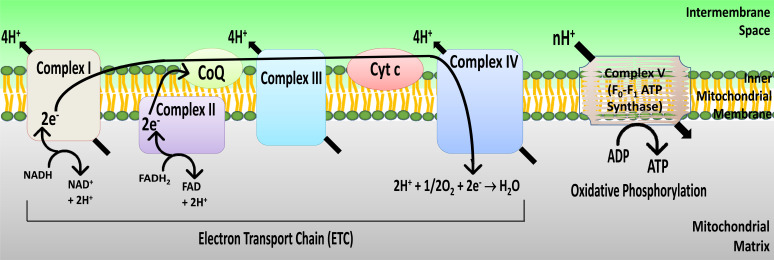
Schematic representation of the electron transport chain (ETC) and oxidative phosphorylation. The ETC consists of a series of oxidoreduction reactions in which the electrons are transferred from the substrate NADH to molecular oxygen through a series of electron carriers and involves oxidoreductase complexes (I to IV) localized in the inner mitochondrial membrane. There is a concomitant release of H^+^ from the mitochondrial matrix into the intermembrane space across the inner mitochondrial membrane, creating a proton gradient which is utilized by the ATP synthase (complex V) for the synthesis of ATP.

The ETC and oxidative phosphorylation serves as the major energy source for neurons, particularly during high-energy demands (Fig. **2**) [3, 114]. As such, brain is very sensitive to the loss of substrates or other impediments to ETC and oxidative phosphorylation. Not surprisingly, activity assays for the ETC complexes have been performed to evaluate brain mitochondrial bioenergetic profiles in several disease states and pathologies. ETC complex activity assays can be performed using both spectrophotometry-based kinetics assessments [40, 115-117] as well as by polarographic measurements of oxygen consumption (section 3.5) [115, 118-120]. For the purpose of the review, we will only focus on the *in organello* spectroscopic assays as they can be easily performed in a time- and cost-efficient manner with the requirement of only the basic reagents and experimental setup.

While a number of slightly different variants exist for spectrophotometric analyses of ETC complexes, the general approach and principle are the same. We have, for instance, evaluated ETC complex activities in both mitochondrial [12] and synaptosomal [11] preparations. Readers are directed to excellent methods-based articles for an extensive overview of the principle and protocols involved [40, 117]. For this review, we focus only on the spectrophotometric assays that have been used in our studies [11, 12].

#### 3.4.1 Complex I (NADH Dehydrogenase; E.C. 1.6.5.3)

The activity of complex I is measured in terms of the reduction of exogenously added potassium ferri(FeIII)cyanide to ferro(FeII)cyanide at 420 nm in the presence of NADH. Rotenone is used to obtain complex I-specific NADH oxidation.

#### 3.4.2 Complex II (Succinate Dehydrogenase; E.C. 1.3.5.1)

The assay for complex II is carried out in a similar manner as the assay for complex I. The difference is in the substrate used, which in this case is succinate. The reductive conversion of the indicator chromogen, potassium ferricyanide to ferrocyanide is again monitored at 420 nm. Pre-treatment of mitochondria with malonate, a competitive complex II inhibitor can be performed as a negative control.

#### 3.4.3 Complex I-III (NADH: Cytochrome c Oxidoreductase)

Complex I-III activity is assessed as the reduction of exogenous cytochrome c which is monitored at 550 nm. The reaction is initiated by the addition of the substrate NADH and in the presence of KCN (an inhibitor of complex IV which oxidizes cytochrome c). Again, only rotenone-sensitive NADH oxidation (and consequent cytochrome c reduction) is taken as the measure of complex I-III activity.

#### 3.4.4 Complex II-III (Succinate: Cytochrome c Oxidoreductase)

The activity of complex II-III is monitored by following the reduction of cytochrome c (exogenous) upon the addition of substrate, succinate. KCN is again used to inhibit complex IV.

#### 3.4.5 Complex IV (Cytochrome c Oxidase; E.C. 1.9.3.1)

For this assay, cytochrome c is first reduced by dithionite. Dithionite-induced reduction of ferri(FeIII)cytochrome (oxidised) to ferro(FeII)cytochrome (reduced) can be conveniently followed as a clear colour change from blood red to pink. Reduced cytochrome c is then quickly purified on a desalting column (*e.g*., Sephadex G-25). Mitochondria-mediated oxidation of exogenous cytochrome c is then evaluated by following the decrease in the absorbance at 550 nm. An air oxidation control for ferrocytochrome c is included for each set.

#### 3.4.6 Complex V (F_0_F_1_ ATP Synthase; E.C. 7.1.2.2)

Oligomycin-sensitive F_0_F_1_ ATP synthase (sometimes called complex V) activity in isolated mitochondria is evaluated by following the hydrolysis of exogenous ATP coupled with the oxidation of exogenous NADH at 340 nm. The detailed procedure is described elsewhere [121-123].

In our studies, we have measured ATP synthesis indirectly utilizing a glucose-hexokinase trap method (excess of hexokinase and glucose-6-phosphate dehydrogenase-coupled enzymes) to ensure a non-rate limiting ADP-regeneration system [11, 12, 41]. Consumption of exogenous inorganic phosphate (P*i*) can then be measured employing a phosphomolybdic acid-ascorbic acid-based assay at 820 nm [11, 12, 124] or by following the reduction of NADP^+^ spectrophotometrically at 340 nm in the extramitochondrial phase [41, 125]. Similarly, Radiolabelled ^[32]^P*i* has also been employed to monitor the residual P*i* in a liquid scintillator counter [126].

The most commonly used method for *in organello* mitochondrial ATP synthesis now-a-days is a bioluminescence-based assay that utilizes exogenous luciferase from firefly (*Photinus pyralis*), an enzyme that oxidizes substrate protein luciferin with the generation of light in an ATP-dependent manner [79, 126]. Since both the enzyme and substrate luciferin are not rate-limiting, bioluminescence measured on a luminometer is directly proportional to ATP concentration. Bioluminescent measurement of ATP levels in isolated mitochondria can also be performed in the presence of different energy substrates [79]. Thus, addition of glutamate and malate have been used to evaluate ATP production with electron flow exclusively through complex I; and succinate and rotenone have been employed to evaluate the contribution of complex II in ATP generation.

### 3.5 Oxygen Consumption and Mitochondrial Coupling

Oxygen consumption is one of the classical end-points that has been evaluated as a measure of mitochondrial function, viability, and oxidative capacity. Oxygen consumption in isolated mitochondria was pioneered by Britton Chance in the 1950s [127]. Recent advances in respirometry have significantly reduced the amount of mitochondria (and hence, the starting tissue) required [128]. The most commonly used method for monitoring mitochondrial oxygen consumption is based on a Clark-type oxygen electrode which polarographically measures oxygen in the solution. Different respiratory states of isolated mitochondria can be measured; oxygen consumption in the presence of substrates alone (state 2), in the presence of substrates and ADP (state 3), and after ADP depletion (state 4). The ratio of oxygen consumption in state 3/ state 4 is the respiratory control ratio (RCR) which is an indicator of mitochondrial coupling (of ATP synthesis and oxygen consumption), and can be conveniently used to calculate mitochondrial integrity and viability [70, 129]. Readers are directed to previously published protocols that have described the method in detail [70, 79, 130].

A fluorescence-based assay employing a MitoXpress^®^ probe (Luxcel Biosciences) has also become popular for high-throughput measurement of oxygen consumption of cells [131, 132] and isolated mitochondrial preparations [69, 133]. The assay is based upon the principle that fluorescence of the MitoXpress^®^ probe is quenched by O_2_ through molecular collision, and hence the amount of fluorescence signal is inversely proportional to the amount of extracellular O_2_ in the sample. A very detailed methodology has been provided elsewhere [134]. Recent studies have also evaluated the rates of oxygen consumption under different energy states using a high throughput Seahorse XF analyser [23, 135]. It should be pointed out here that barring the MitoXpress^®^-based fluorimetric assay, all other assays require specialized equipment (and accessories).

### 3.6 Tricarboxylic Acid Cycle (TCA) Enzyme Activities

The TCA or Krebs cycle is the central pathway of metabolism and is involved in the oxidation of acetyl coenzyme A derivatives obtained by the catabolism of carbohydrates, amino acids, and fatty acids. Pyruvate dehydrogenase which generates acetyl coenzyme A is a key link between glycolysis and the TCA cycle. TCA enzymes are localized in the mitochondrial matrix wherein they are organized as a supramolecular complex, called the Krebs metabolon. However, some of the TCA enzymes (*e.g*., aconitase, fumarase, malate dehydrogenase) have cytosolic counterparts that perform extramitochondrial functions. Of note, TCA enzymes may have additional moonlighting functions such as stabilization of mitochondrial DNA (mtDNA) and mitochondrial mRNA translation, *etc*. [136].

Activity assays of pyruvate dehydrogenase complex and TCA cycle enzymes can be performed easily *in organello* in isolated mitochondrial preparations using simple spectrophotometric protocols [137]. We have previously listed the activity assays for fumarase (section 2.2.7.3), CS (section 2.2.7.2), and SDH (section 3.4.2). Readers are directed to detailed activity assay protocols for pyruvate dehydrogenase and other TCA enzymes (aconitase, isocitrate dehydrogenase, α-ketoglutarate dehydrogenase, succinyl CoA synthase, and malate dehydrogenase) published elsewhere [138].

## 4 OXIDATIVE DAMAGE AND REDOX HOMEOSTASIS

Mitochondrial ETC and several other oxidases are the major sources of reactive oxygen species (ROS) such as superoxide ion (O_2_^.-^), hydrogen peroxide (H_2_O_2_), and hydroxyl radical (^•^OH). Unsurprisingly, there are a multitude of sophisticated endogenous mitochondrial antioxidant mechanisms, ranging from low molecular weight antioxidants like glutathione (GSH) to detoxifying enzymes such as superoxide dismutases (SODs), catalase, and peroxidases, acting as primary lines of defence [139]. Nevertheless, dysregulation of these endogenous antioxidants and oxidative damage to proteins and lipids appears to be a chief feature of several neuropathologies [140, 141]. Hence, assessment of redox signalling, oxidative damage, and antioxidant capacity of *ex vivo* mitochondrial preparations has been a key focus area of research evaluating the role of mitochondria in brain diseases. This section attempts to briefly summarize some of the *in organello* biochemical assays used for evaluation of the major indices of mitochondrial redox signalling.

### 4.1 Reactive Oxygen (ROS) and Nitrogen (RNS) Species Production

#### 4.1.1 DCF Assay

The most widely used fluorogenic probe to measure ROS/RNS production is dichlorodihydrofluorescein diacetate (DCFH-DA). In presence of ROS/RNS, non-fluorescent DCFH-DA is converted to a brightly fluorescent dichlorofluorescein (DCF) [142]. DCF fluorescence can be monitored in isolated mitochondrial samples, both at basal and energized states as described in previous studies, either as a kinetic assay or as an end-point assay [11, 12, 54].

#### 4.1.2 Superoxide Anion (O_2_^.^^-^)/H_2_O_2_ Production

ROS production by the ETC in mitochondria occurs predominantly by the premature leak of electrons from complexes I, II, and III, resulting in partial reduction of oxygen to superoxide (O_2_^.^^-^) which is then dismutated to hydrogen peroxide (H_2_O_2_) [143]. Mitochondrial production of H_2_O_2_ is most commonly evaluated using Amplex Red [95, 142]. In the presence of mitochondrial H_2_O_2_ and exogenous horseradish peroxidase, Amplex Red is oxidized to resorufin which can be monitored fluorimetrically [144, 145]. *In organello* mitochondrial O_2_^.^^-^ production can also be measured spectrophotometrically by following the reduction of exogenously added oxidized-cytochrome c (Fe^3+^-cyt c) [85, 146] or epinephrine [147].

#### 4.1.3 Nitric Oxide (NO^•^) End Products

Nitrosative species such as NO^•^ are known to be detrimental to mitochondrial functions, particularly the respiratory proteins [148]. In our studies, we have used a spectrophotometric assay for evaluating NO^•^ end products (nitrites and nitrates), employing Griess reagent [11, 12]. A spectrophotometric assay based on the oxidation of exogenous oxyhemoglobin (HbO_2_) for NO production in mitochondria (activity assay of mitochondrial nitric oxide synthase; mtNOS) has also been employed in literature [149, 150].

### 4.2 Oxidative Damage to Lipids and Proteins

Simple *in organello* spectrophotometric assays for assessment of oxidative damage to lipids (peroxidation) and proteins (carbonylation and thiol oxidation) have been carried out in our studies for both isolated mitochondria [12] and synaptosomes [11].

### 4.3 Mitochondrial Antioxidant Potential

#### 4.3.1 General Antioxidant Capacity

*In organello* evaluation of the free radical scavenging capacity of mitochondria has been performed by us utilizing 2,2’-azino-bis(3-ethylbenzothiazoline-6-sulfonic acid) or ABTS dye [11, 12, 151]. The radical form of ABTS (denoted as ABTS^+•^) is generated in the presence of (potassium) persulfate and has absorption maxima at 660, 734, and 820 nm. As reduction of ABTS^+•^ results in its decolourization, mitochondrial scavenging of ABTS^+•^ can be evaluated at 734 nm. The degree of antioxidant capacity of ABTS^+•^ radical scavenging can be quantified in comparison with a standard curve of Trolox (a water-soluble analogue of vitamin E), and expressed in terms of Trolox equivalent antioxidant capacity (TEAC) [152]. Similar assay involving quenching of peroxy radicals from azo initiators, such as 2, 2’-azobis(2-amidinopropane) dihydrochloride or ABAP (Total radical-trapping antioxidant parameter assay or TRAP; [153]) and 2, 2’-azobis(2-methylpropionamidine) dihydrochloride or AAPH (Oxygen radical absorbance capacity or ORAC; [154]) have also been employed for measuring the endogenous antioxidant ability of isolated mitochondria.

Another commonly used method to evaluate mitochondrial antioxidant power is the ferric reducing antioxidant power (FRAP) assay which is based upon the ability to reduce ferric compounds (such as Fe^III^-tripyridyltriazine) to ferrous counterparts [151, 155].

#### 4.3.2 Glutathione (GSH) Antioxidant Signalling

GSH is the most abundant small molecule thiol present in the brain [156, 157] and is the major mitochondrial antioxidant [158]. Scavenging of oxidative species like H_2_O_2_ and other hydroperoxides involves oxidation of GSH to GSSG by the action of glutathione peroxidase (GPx). Recycling of GSSG back to its reduced GSH form is carried out by glutathione reductase (GR). Yet another mechanism of detoxification of oxidizing species is their conjugation with GSH catalysed by the enzyme glutathione S-transferase (GST) [157, 159]. Assessment of the endogenous mitochondrial GSH-mediated antioxidant activity hence requires assessment of the levels of reduced GSH and activities of the GSH-related enzymes; GPx, GR, and GST. The spectroscopy based *in organello* protocols for each of these assays have been described in detail in our previous publication [11].

#### 4.3.3 Superoxide Dismutase (SOD) Activity

Several variants of the assays for SOD activity have been efficiently employed in the literature. These are based upon detection of superoxides by using dyes such as nitroblue tetrazolium (NBT; [160]), adrenaline [161], or sulphanilamide [162]. One of the most commonly employed assays that deserve a special mention here is the spectrophotometric assay employing a xanthine-xanthine oxidase system to generate superoxide ion and oxidized cytochrome c as the superoxide-trapping detection system. The methodology for this assay is described in detail elsewhere [163].

## 5 MITOCHONDRIAL PROTEIN TRANSLATION

While over 99% of the mitochondrial proteins are encoded by the nuclear DNA and translated in the cytosolic ribosomes, mtDNA encodes 37 genes, of which 13 code for mitochondrial proteins which are essential components of the ETC, and the rest code for rRNAs and tRNAs needed for their translation [164]. Recent studies are establishing the critical roles of local *de novo* translation of mitochondrial proteins in maintaining neuronal function, particularly synaptic physiology and plasticity [164, 165]. In this regard, *in organello* approaches to study protein translation in isolated mitochondria have been successfully used with yeast, plant, and mammalian cells [166]. It should be noted that biochemically isolated mitochondrial preparations are often contaminated, at least to some degree with microsomes and other membrane fractions studded with cytosolic ribosomes. Hence, it is essential to use blockers of cytoplasmic protein synthesis, such as emetine and cycloheximide to study mitochondria-specific protein translation. Correspondingly, our assays of synapse-specific protein translation in isolated synaptic terminals are indispensable without a mitochondrial protein synthesis blocker, such as chloramphenicol because of the presence of intrasynaptic mitochondria and free mitochondrial contaminants [167-169].

Local *de novo* protein translation in isolated mitochondria has been assayed utilizing [^35^S]-radiolabelled cysteine and methionine, followed by either gel autoradiography or radioactivity counting [166, 170-173]. In the literature, we only found references for radioactivity-based protocols for *in organello* mitochondrial protein translation. It will be interesting to follow the development of alternative non-radioactive methods for the evaluation of mitochondrial protein synthesis, such as those based on puromycin [167, 169], an aminoacyl-tRNA analogue that has similar action on cytosolic and mitochondrial protein translation [174]. Intriguingly, puromycin-mediated assessment of mitochondrial translation has been carried out in an adherent cell culture system with the concomitant presence of emetine, a blocker of cytosolic protein synthesis [175].

## 6 PROTEOMIC CHARACTERIZATION

Proteomic characterization is the most obvious and most widely employed application of isolated mitochondria, both in terms of the global proteomic characterization of mitochondrial proteins and their post-translational modifications. Both gel-based and gel-free comparative mitochondrial proteomics methodologies have been widely-used to evaluate the quantitative and qualitative differences in mitochondrial proteome as a function of subcellular localization (*e.g*., synaptic *versus* non-synaptic mitochondria; [9]), developmental stage (*e.g*., embryonic *versus* postnatal, or young *versus* aged [176]), disease conditions (*e.g*., in Alzheimer’s disease [177]; and multiple sclerosis [178]) as well as treatment with therapeutic agents (*e.g*., bis(7)-tarcine [179]; and simvastatin [180]). Since excellent recent review articles have delineated both the techniques of mitochondrial proteomics as well as their contribution to our understanding the of pathology of brain diseases [177, 181-190], we will not discuss this topic further.

## 7 NORMALIZATION OF MITOCHONDRIAL CONTENT

Normalization for *in organello* assays is essential in order to evaluate the actual inherent mitochondrial properties, independent of the differences in the mitochondrial content of different preparations. There are several ways to do this. The simplest of these are normalizing mitochondrial protein content using the established protein quantitation assays such as Bradford or bicinchoninic acid (BCA) methods. However, this method is not specific for mitochondria (*i.e*. it does not differentiate between the mitochondrial content and the other minute impurities that may be present in the preparation) and hence may be erroneous. A better normalization control is the activity of CS [79, 191]. In our opinion, normalization at both levels (total protein content and citrate synthase activity) should be performed for functional analyses of mitochondria.

## CONCLUSION

While studies have provided some evidences for the utility of *ex vivo* mitochondrial preparations in the characterization of the physiology and pathophysiology of neuronal mitochondria, it is clear that the use of these preparations has been under-utilized in neurobiology research.

In particular, research on the synaptic mitochondrial pool is lagging behind, in spite of the knowledge that synapses are the major sites of interneuronal communication and substrates for almost all higher-order functions and their dysfunction is a common and primary pathogenic event in brain pathologies across the whole development span, from early-life insults to ageing-related neurodegeneration. Given the critical roles of synaptic mitochondria in neurotransmission, it is surprising that only a few studies have been directed against evaluating their functions specifically. In this regard, biochemically isolated synaptic mitochondria seem to be the most important step forward to evaluate their function (and dysfunction) in relation to synaptic physiology and brain functions.

The purpose of the review is to solely summarize the methodologies and protocols of mitochondrial research, specifically using isolated brain mitochondrial samples (Table **1**). However, in doing so, the hope is that it will help neuroscientists to identify and appreciate the utilities of these biochemical fractions and encourage them to employ their expertise in understanding the finer complexities of mitochondrial (and synaptic) pathology in healthy and diseased brain states. We also hope that through this review, researchers will be motivated to identify the gaps and device novel assays to evaluate mitochondrial (patho)physiology. One can only hope for an escalation in the employment of *ex vivo* mitochondria from cellular and animal models and human subjects to further delineate the contribution of mitochondria to brain function and dysfunction, and to evaluate the efficiency of potential therapeutic agents/strategies in prevention and amelioration of multiple neuropathologies.

## Figures and Tables

**Table 1:** Summary of the *in organello* assays for mitochondrial quality assessment, viability and functions.

**S. No.**	**Assay Name**	**Assay Type**	**Information Provided**
**Quality Control Procedure**			
1.	Electron microscopy	Transmission and Scanning electron microscope (TEM and SEM)	Mitochondrial enrichment/yield and morphological integrity
2.	Mitochondrial stains	Spectroscopy-, Flow cytometry-, or Fluorescence microscopy-based	Mitochondrial enrichment and integrity
3.	Marker proteins	Immunoblotting	Enrichment
4.	Lactate dehydrogenase activity assay	Spectroscopy-based	Mitochondrial enrichment/yield and integrity
5.	Cytochrome c release	Immunoblotting or spectroscopy-based	Mitochondrial enrichment/yield and integrity
6.	Oxidation of exogenous ascorbate/ cytochrome c	Spectroscopy-based or polarographic assay	Mitochondrial intactness
7.	Succinate dehydrogenase (Complex II) activity assay	Spectroscopy-based	Mitochondrial enrichment/yield and integrity
8.	Citrate synthase activity assay	Spectroscopy-based	Mitochondrial enrichment/yield and integrity, and as a normalization control
9.	Fumarase activity assay	Spectroscopy-based	Mitochondrial integrity
10.	Adenylate kinase activity assay	Spectroscopy-based	Mitochondrial integrity
11.	Glutamate dehydrogenase activity assay	Spectroscopy-based	Mitochondrial integrity
12.	Calcium buffering capacity	Fluorimetric	Mitochondrial integrity
**Functional and Bioenergetic Assays**			
1.	Mitochondrial membrane potential (cationic fluorescent dyes)	Fluorimetric, fluorescence spectroscopy- or cell cytometry-based	Mitochondrial integrity, viability and bioenergetic capacity
2.	Ca^2+^-induced swelling (mitochondrial permeability transition)	Light scattering (spectroscopy)-based	Mitochondrial integrity, viability and bioenergetic capacity
3.	Calcium buffering capacity (Ca sensitive fluorescent dyes)	Fluorescence spectroscopy-based	Mitochondrial integrity and viability
4.	Electron transport chain complexes activity assays - Complex I - Complex II - Complex I-III - Complex II-III - Complex IV - Complex V	Spectroscopy-based, polarographic, or bioluminescence-based assay	Mitochondrial viability and bioenergetic capacity
5.	Oxygen consumption	Polarographic or fluorescence spectroscopy-based	Mitochondrial viability and bioenergetic capacity
6.	Tricarboxylic acid cycle enzyme activity assays	Spectroscopy-based	Mitochondrial viability and bioenergetic capacity
**Assays for Oxidative Damage and Redox Homeostasis**			
1.	Dichlorofluorescein (DCF) assay	Fluorescence spectroscopy-based	Amounts of oxidative species
2.	Superoxide/peroxide production	Spectroscopy- or fluorescence spectroscopy-based	Production of superoxide anion and hydrogen peroxide
3.	Griess assay	Spectroscopy-based	Amounts of nitric oxide end products
4.	Lipid peroxidation	Spectroscopy-based	Lipid oxidation
5.	Protein carbonylation	Immunoblotting or spectroscopy-based	Protein oxidative damage
6.	Protein thiol oxidation	Spectroscopy-based	Protein oxidative damage
7.	Antioxidant capacity	Spectroscopy-based	Free radical scavenging
8.	Glutathione (GSH) signalling - Reduced GSH levels - Glutathione reductase - Glutathione peroxidase - Glutathione S-transferase	Spectroscopy- or fluorescence spectroscopy-based	Antioxidant GSH signalling
9.	Activity assay for superoxide dismutase	Spectroscopy- or fluorescence spectroscopy-based	Superoxide scavenging
**Protein Translation Assays**			
1.	Radiolabeled cysteine/methionine incorporation assay	Liquid scintillation or gel autoradiograhy	*De novo* protein translation
2.	Puromycin incorporation assay	Immunoblotting	*De novo* protein translation

## LIST OF ABBREVIATIONS

AAPH: 2, 2’-azobis(2-methylpropionamidine) dihydrochloride
ABAP: 2,2’-azobis(2-amidinopropane) dihydrochloride
ABTS: 2,2’-azino-bis(3-ethylbenzothiazoline-6-sulfonic acid)
AK: Adenylate Kinase
BCA: Bicinchoninic Acid
CMP: Crude Mitochondrial Pellet
CS: Citrate Synthase
DCF: Dichlorofluorescein
DCFH-DA: Dichlorodihydrofluorescein Diacetate
DMSO: Dimethyl Sulfoxide
DTNB: 5,5’-dithiobis(2-nitrobenzoic acid)
EM: Electron Microscopy
ETC: Electron Transport Chain
FACS: Fluorescence-activated Cell Sorting
FRAP: Ferric Reducing Antioxidant Power
GDH: Glutamate Dehydrogenase
GPx: Glutathione Peroxidase
GR: Glutathione Reductase
GSH: Glutathione, Reduced
GSSG: Glutathione, Oxidized
GST: Glutathione S-transferase
HbO_2_: Oxyhemoglobin
IMM: Inner Mitochondrial Membrane
JC-1: 5,5’,6,6’,-tetrachloro-1,1’,3,3’-tetraethyl-benzimidazolylcarbocyanine iodide
LDH: Lactate Dehydrogenase
MAM: Mitochondria-associated Membranes
MMP (Δψ_m_): Mitochondrial Membrane Potential
mPT: Mitochondrial Permeability Transition
mPTP: Mitochondrial Permeability Transition Pore
mtDNA: Mitochondrial DNA
mtNOS: Mitochondrial Nitric Oxide Synthase
NBT: Nitroblue Tetrazolium
NO: Nitric Oxide
NP: Nuclear Pellet
OMM: Outer Mitochondrial Membrane
ORAC: Oxygen Radical Absorbance Capacity
PMS: Post Mitochondrial Supernatant
PNS: Post Nuclear Supernatant
RCR: Respiratory Control Ratio
RNS: Reactive Nitrogen Species
ROS: Reactive Oxygen Species
SDH: Succinate Dehydrogenase
SM: Synaptic Mitochondria
SOD: Superoxide Dismutase
TCA: Tricarboxylic Acid Cycle
TEAC: Trolox Equivalent Antioxidant Capacity
TMPD: N,N,N’,N’-tetramethyl-p-phenylenedia-mine
TNB^-^: 5’-Thionitrobenzoate Anion
TOM: Translocase of the Outer Membrane
TRAP: Total Radical Trapping Antioxidant Parameter Assay
VDAC: Voltage-dependent Anion-selective Channel
